# MiR-4653-3p and its target gene FRS2 are prognostic biomarkers for hormone receptor positive breast cancer patients receiving tamoxifen as adjuvant endocrine therapy

**DOI:** 10.18632/oncotarget.11278

**Published:** 2016-08-13

**Authors:** XiaoRong Zhong, GuiQin Xie, Zhang Zhang, Zhu Wang, Yu Wang, YanPing Wang, Yan Qiu, Li Li, Hong Bu, JiaYuan Li, Hong Zheng

**Affiliations:** ^1^ Laboratory of Molecular Diagnosis of Cancer, State Key Laboratory of Biotherapy, National Collaborative Innovation Center for Biotherapy, West China Hospital, Sichuan University, Chengdu 610041, P. R. China; ^2^ Department of Pathology, West China Hospital, Sichuan University, Chengdu 610041, P. R. China; ^3^ Laboratory of Pathology, West China Hospital, Sichuan University, Chengdu 610041, P. R. China; ^4^ Department of Epidemiology and Bio-Statistics, West China School of Public Health, Sichuan University, Chengdu 610041, P. R. China; ^5^ Cancer Center, West China Hospital, Sichuan University, Chengdu 610041, P. R. China

**Keywords:** miR-4653-3p, FRS2, breast cancer, tamoxifen resistant, prognostic biomarker

## Abstract

Long-term tamoxifen treatment significantly improves the survival of hormone receptor-positive (HR+) breast cancer (BC) patients. However, tamoxifen resistance remains a challenge. We aimed to identify prognostic biomarkers for tamoxifen resistance and reveal the underlying mechanism. From March 2001 to September 2013, 400 HR+ BC women (stage I~III) were treated with adjuvant tamoxifen for 5 years or until relapse in West China Hospital. We included a discovery set of 6 patients who were refractory to tamoxifen, and a validation cohort of 88 patients including 35 cases with relapse. In the discovery set, microRNA microarray showed that miR-4653-3p decreased in recurrent/metastatic lesions compared to the matched primary lesions. In the validation cohort, real-time RT-PCR demonstrated that, following tamoxifen treatment, miR-4653-3p overexpression in the primary tumors decreased the risk of relapse (adjusted hazard ratio [HR] = 0.17, 95% confidence interval [CI] = 0.05~0.57, *P* = 0.004). Conversely, high expression of FRS2, the key adaptor protein required by FGFR signaling, predicted poor disease-free survival (DFS) (adjusted HR = 2.70, 95% CI = 1.11~6.56, *P* = 0.03). MiR-4653-3p down regulated FRS2 by binding to its 3′ untranslated region. Either overexpressing miR-4653-3p or attenuating FRS2 expression could restore TAM sensitivity in two tamoxifen-resistant BC cell lines. In conclusion, high miR-4653-3p level was the potential predictor for favorable DFS, while FRS2 overexpression was potential high-risk factor for relapse in HR+ BC patients receiving TAM adjuvant therapy. FGFR/FRS2 signaling might be a promising target for reversing tamoxifen resistance.

## INTRODUCTION

Breast cancer is the most common cancer and the fifth leading cause of cancer-related deaths among women in China [1]. Approximately 70% of breast cancer patients expressed estrogen receptor alpha (ERα) [2]. This population is eligible for endocrine therapy, which includes 3 categories: selective ER modulators (SERMs) such as tamoxifen (TAM), selective ER down regulators, and aromatase inhibitors (AIs). Among ER-positive (ER+), early breast cancer (EBC) patients, 5-year of adjuvant TAM treatment could reduce the 10-year recurrence rate and 15-year mortality by about one third [3]. The Adjuvant TAM: Longer Against Shorter trial (ATLAS) showed that prolonged treatment of TAM for 10 years further reduced recurrence and mortality in patients with ER+ EBC [4]. Despite the effectiveness of TAM treatment, 21%~25% of patients will acquire resistance to TAM about 5~14 years after first diagnosis [4]. Hence, the discovery of prognostic biomarkers for TAM will facilitate the identification of patient who will benefit most from TAM.

MicroRNAs (miRNAs) are short, single-stranded non-coding RNAs. MiRNAs bind to 3′ untranslated region (3′ UTR) of mRNAs and subsequently inhibit protein translation or degrade the transcripts [5]. Studies using TAM-resistant cell models showed that miRNAs are important upstream regulator of TAM resistance, via modification of ER expression and ERα signaling-growth factor receptors signaling, cell cycle, apoptosis/cell survival signaling and epithelial-to-mesenchymal transition [6]. A few available clinical studies indicated that 7 miRNAs (miR-10a, miR-26, miR-30c, miR-126a, miR-210, miR-342, and miR-519a) were associated with the outcome of TAM treatment [7–13]. Besides, miRNAs are stable in long-term stored formalin-fixed paraffin-embedded (FFPE) tissues [14] and blood samples [15]. Thus, miRNAs are promising biomarkers for predicting TAM efficacy.

Abnormal activation of Fibroblast growth factor/Fibroblast growth factor receptor (FGF/FGFR) signaling pathways is an important mechanism underlying endocrine resistance. MCF-7 breast cancer cells overexpressing FGF- 1 formed metastatic tumors in TAM-treated nude mice [16]. FGFR3 expression increased in TAM resistant breast tumors. Activation of FGFR3 reduced the sensitivity to tamoxifen via activation of MAPK and PI3K pathways in MCF7 cells [17]. Moreover, FGFRs might be an effective target for anti-tumor therapy (e.g. dovitinib, a broad-range tyrosine kinase inhibitor targeting FGFR1/2/3 and other receptors [18]). Fibroblast growth factor receptor substrate 2 (FRS2, also known as FRS2alpha) is a member of the adaptor/scaffold protein family. It was considered as an essential “conning center” in FGFR signaling [19]. FSR2 binds through its N-terminal phosphotyrosine-binding domain (PTB) to FGFR, resulting in phosphorylation of multiple tyrosine residues of FRS2 and subsequent activation of the downstream signaling (e.g., RAS/MAPK/ERK, PI3K/AKT/mTOR) and ubiquitination/degradation pathways [19]. FRS2 acts as an oncogene in a variety of cancers, which regulates tumor cell differentiation, proliferation, and tumorigenesis [20]. Inhibition of FRS2 could block the FGFR signaling and downstream biological functions [21–23]. However, the roles of FRS2 in endocrine resistance in breast cancer are unclear.

In the present study, we aimed to find prognostic biomarkers for HR+ breast cancer patients receiving tamoxifen as adjuvant endocrine therapy. Since paired primary and recurrent/metastatic (R/M) lesions have the same genetic background, differentially expressed miRNAs and genes between those lesions are possibly correlated with treatment failure. Those abnormal signals or biomarkers might also exist originally in the primary tumors. We began with a comparison of miRNA profile in paired primary and R/M lesions from 6 HR+ BC patients who were refractory to TAM. The baseline level of the identified miRNA and its target gene in primary tumors were detected in a cohort of 88 patients. Their potential to predict disease-free survival (DFS) following TAM adjuvant therapy was estimated. Functional experiments using TAM-resistant cell models further revealed the underlying mechanisms of TAM resistance via activation of FGFR/FRS2 signaling.

## RESULTS

### Differential expressed miRNA profiles of primary and recurrent/metastatic lesions

We used Exiqon miRCURY™ LNA Array (v.18.0) to compare the miRNA expression profiles in paired primary and R/M lesions from the discovery set of 6 breast cancer patients who relapsed after TAM treatment. Fold change (FC) was calculated as the mean ratio of normalized miRNA levels in R/M lesions to matched primary lesions. We defined FC ≥ 2 or < 0.5 with a *P* value < 0.05 (paired *t*-test or Wilcoxon signed ranks test) as differential expression. Out of the 1921 human miRNAs tested, 28 miRNAs were significantly downregulated in R/M lesions (FC range: 0.27~0.50), and 54 miRNAs were significantly upregulated (FC range: 2.00~13.63) (Supplementary Table S1). We deposited the raw data of miRNA miroarray in NCBI's Gene Expression Omnibus (GEO) [24], and are accessible through GEO Series accession number GSE83292 (https://www.ncbi.nlm.nih.gov/geo/query/acc.cgi?acc=GSE83292).

**Figure 1:**
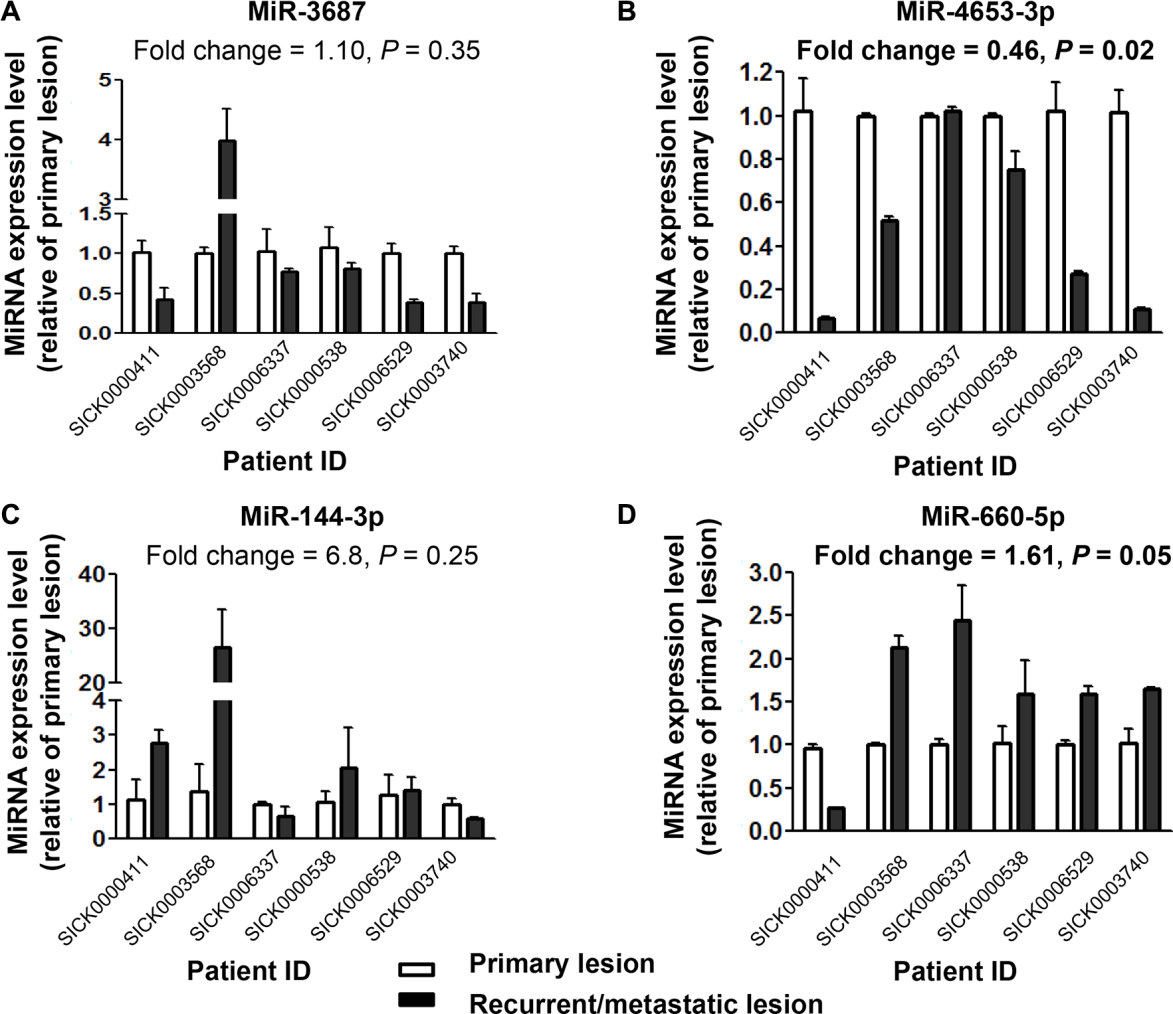
Downregulation of miR-4653-3p and upregulation of miR-660-5p confirmed in recurrent/metastatic lesion, compared to their matched primary lesion in tamoxifen-resistant patients Total RNA was extracted from tumor tissues of recurrent/metastatic (R/M) lesions and their matched primary lesions of 6 TAM-resistant patients from the discovery set. Real-time RT-PCR was performed for evaluating levels of (**A**) miR-3687, (**B**) miR-4653-3p, (**C**) miR-144-3p and (**D**) miR-660-5p. RNU6B served as an internal control for normalization purpose. Fold change was calculated as the mean ratio of normalized miRNA levels in R/M lesions to matched primary lesions. *P* values were calculated using paired two-sided *t* test or Wilcoxon signed ranks test (related samples) as appropriate.

MiR-3687 (FC = 0.27, *P* = 0.006) and miR-4653- 3p (FC = 0.28, *P* = 0.03) were the most downregulated miRNAs in R/M lesions; while miR-144-3p (FC = 10.27, *P* = 0.04) was one of the most upregulated miRNAs. MiR-660-5p, upregulated in R/M lesions here (FC = 3.70, *P* = 0.004), has been reported as a potential prognostic biomarker for overall survival of breast cancer previously [25]. Downregulation of miR-4653-3p (FC = 0.46, *P* = 0.02) and upregulation of miR-660-5p (FC = 1.61, *P* = 0.05) in R/M lesions were successfully confirmed by real-time RT-PCR (Figure 1B, 1D). MiR-4653-3p was then chosen to be the candidate for prognostic biomarker, considering prominent FC, good sensitivity for real-time RT-PCR detection (Ct value range: 20~26), and novelty.

### High level of miR-4653-3p predicted better DFS following TAM treatment

**Figure 2:**
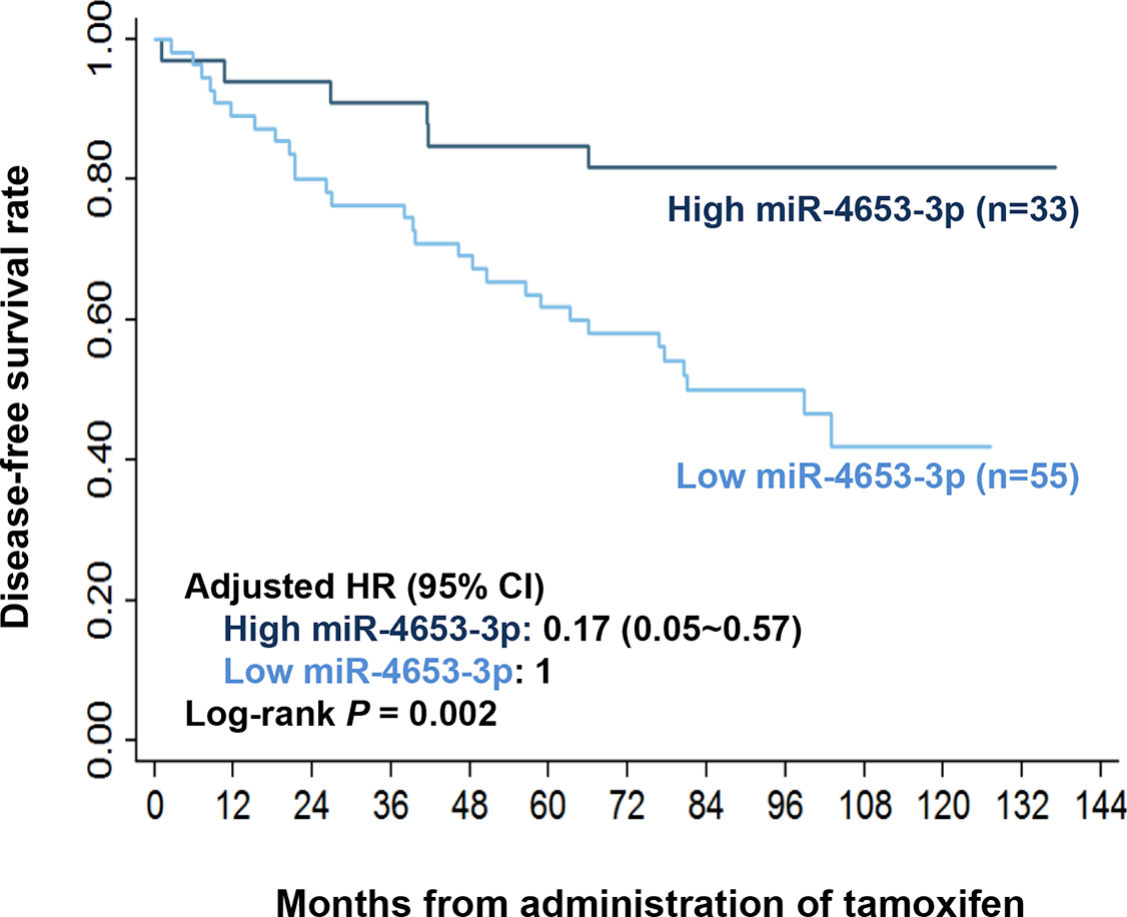
High miR-4653-3p level was associated with better disease-free survival following tamoxifen in breast cancer patients MiR-4653-3p levels in primary tumor tissues from the validation cohort were detected by real-time RT-PCR. Estimated Kaplan-Meier disease-free survival curves of patients with high (≥ 0.43) and low (< 0.43) expression of miR-4653-3p were compared. The optimal cutoff value for miRNA-4653-3p level was determined by ROC curve. Hazard ratio (HR) was adjusted by age at diagnosis, tumor size, lymph node involvement, Ki67 expression, HER2 status, menopause status when receiving tamoxifen and adjuvant chemotherapy. *P* values were calculated using a log-rank analysis.

We hypothesized that miR-4653-3p, which downregulated in R/M lesions during relapse process, might also have different baseline levels in primary tumors and associated with disease prognosis. To test the hypothesis, we used real-time RT-PCR to evaluate its expression in the primary tumors from the validation cohort of 88 cases. Relapse occurred in 35 patients, 26 of which occurred within 5 years following TAM treatment. To define the high and low miR-4653-3p level, we chose a cutoff of 0.43 on the receiver operating characteristics (ROC) curve for distinguishing patients who were likely to relapse within 5 years. At this cutoff value, the area under ROC curve [AUC] was 0.65 (95% confidence interval [CI] = 0.53~0.77, *P* = 0.03) with a sensitivity of 80.8% and specificity of 45.2%. The Kaplan-Meier plot showed that 5-year DFS rate following TAM treatment was significantly higher in high miR-4653-3p expression group (≥ 0.43, *n* = 33) than low expression group (< 0.43, *n* = 55) (5 year DFS rate: 84.8% ± 6.2% vs. 61.8% ± 6.6%; log-rank *P* = 0.002; Figure 2). Moreover, in the univariate analysis, high miR-4653-3p level significantly reduced the risk of relapse by 72% (hazard ratio [HR] = 0.28, 95% CI = 0.12~0.68, *P* = 0.005; Table 1). After adjusting seven prognostic factors (age at diagnosis, tumor size, lymph node involvement, Ki67 expression, HER2 status, menopause status when receiving TAM and adjuvant chemotherapy), the statistical difference remained (adjusted HR = 0.17, 95% CI = 0.05~0.57, *P* = 0.004; Table 1, Figure 2). Besides, lymph node involvement and positive HER2 status also contributed to poor DFS.

**Table 1:** High level of miR-4653-3p was associated with better disease-free survival in the validation cohort

Parameter	#Cases (events)	Unadjusted HR (95% CI)^a^	*P*	Adjusted HR (95% CI)^b^	*P*
**miR-4653-3p relative expression**					
High (≥ 0.43)	33 (6)	0.28 (0.12~0.68)	**0.005**	0.17 (0.05~0.57)	**0.004**
Low (< 0.43)	55 (29)	1		1	
Age at diagnosis	88 (35)	0.97 (0.93~1.02)	0.22	0.98 (0.91~1.05)	0.56
Clincal stage					
I	17 (2)	1			
II	41 (14)	3.54 (0.8~15.6)	0.09		
III	27 (18)	9.42 (2.17~40.85)	**0.003**		
Tumor size					
T ≤ 2 cm	21 (4)	1		1	
2 cm < T ≤ 5 cm	53 (23)	2.69 (0.93~7.81)	0.07	0.7 (0.17~2.85)	0.62
T > 5 cm	11 (7)	5.41 (1.57~18.62)	**0.007**	0.69 (0.13~3.61)	0.66
Lymph node involvement					
Negative	38 (6)	1		1	
Positive	49 (29)	5.22 (2.16~12.61)	**0.0002**	11.58 (2.87~46.67)	**0.001**
Tumor grade					
I/II	35 (11)	1			
III	48 (23)	1.75 (0.85~3.6)	0.13		
Molecular subtype					
Luminal A	24 (9)	1			
Luminal B	49 (18)	1.07 (0.48~2.39)	0.86		
Ki67					
< 14%	33 (12)	1		1	
≥ 14%	49 (17)	0.97 (0.46~2.03)	0.93	1.67 (0.62~4.44)	0.31
HER2					
Negative	76 (27)	1		1	
Positive	5 (3)	2.43 (0.73~8.03)	0.15	5.77 (1.19~28.08)	**0.03**
Menopause when receiving tamoxifen					
Premenopause	63 (23)	1		1	
Postmenopause	23 (10)	1.06 (0.50~2.24)	0.87	0.99 (0.28~3.47)	0.99
Adjuvant chemotherapy					
Yes	84 (35)	1		1	
No	4 (0)	0.04 (0~20.48)	0.32	0.00	0.98

a Univariate Cox proportional hazards regression models. Unknown data were not included in the analysis.

b HR was adjusted by the following confounders: age at diagnosis, tumor size, lymph node involvement, Ki67 expression, HER2 status, menopause status when receiving tamoxifen and adjuvant chemotherapy.

### Functional annotation of miR-4653-3p-targeted genes

MiR-4653-3p-targeted genes were predicted using the following algorithms: miRDB (MirTarget2, http://mirdb.org/miRDB/), TargetScan (TargetScan7.1, http://www.targetscan.org/) and DIANA (MICROT MicroT-CDS, http://diana.imis.athena-innovation.gr/DianaTools/index.php). There were 79 target genes present in all the 3 predictions (Supplementary Table S2). Gene-enrichment and functional annotation analysis was performed by using Functional Annotation Tool (DAVID Bioinformatics Resources 6.7, NIAID/NIH, http://david.abcc.ncifcrf.gov/) [26]. These 79 genes were significantly enriched into 25 Gene Ontology (GO) Terms :12 for biological process, 8 for cellular component, and 5 for molecular function (Table 2). Notably, 3 enriched GO terms were relevant with growth factor receptor signaling: regulation of MAP kinase activity, regulation of protein kinase activity, regulation of kinase activity. DRD1, PDGFB, PPP2CA, PRKAA1 and FRS2 were involved in the mentioned 3 terms. Among them, FRS2 is the essential linker between FGFRs and their downstream RAS/MAPK/ERK and PI3K/AKT/mTOR signaling [19]. Two sites located in base pairs 84~90 and 2213~2219 on the 3′UTR of FRS2 mRNA were predicted to be complimentary to miR- 4653-3p. Thus, we speculated that FRS2 might be one of the most important target genes of miR-4653-3p that contributes to TAM resistance.

**Table 2:** Gene-enrichment and functional annotation analysis of miR-4653-3p-targeted genes^a^

GO Term ID	Enriched Term	Gene Counts	*P*^b^	Target Genes
**Biological process (database: GOTERM_BP_FAT)**				
GO:0006461	protein complex assembly	7	0.012	TAF1, TAF4, IPO5, TRIM27, AHCTF1, ITPR3, SEPT7
GO:0070271	protein complex biogenesis	7	0.012	TAF1, TAF4, IPO5, TRIM27, AHCTF1, ITPR3, SEPT7
GO:0065003	macromolecular complex assembly	7	0.041	TAF1, TAF4, IPO5, TRIM27, AHCTF1, ITPR3, SEPT7
GO:0043405	regulation of MAP kinase activity	4	0.017	PDGFB, PPP2CA, PRKAA1, FRS2
GO:0045859	regulation of protein kinase activity	5	0.043	DRD1, PDGFB, PPP2CA, PRKAA1, FRS2
GO:0043549	regulation of kinase activity	5	0.048	DRD1, PDGFB, PPP2CA, PRKAA1, FRS2
GO:0008104	protein localization	9	0.018	AP1S3, DRD1, DERL2, GOLT1B, RAB4A, DMD, NUP50, IPO5, SRGN
GO:0050808	synapse organization	3	0.023	DRD1, PCDHB3, NFASC
GO:0043062	extracellular structure organization	4	0.025	DRD1, PCDHB3, COL3A1, NFASC
GO:0060260	regulation of transcription initiation from RNA polymerase II promoter	2	0.027	TAF1, AHR
GO:0019725	cellular homeostasis	6	0.033	DRD1, TXNDC16, DMD, STIM2, PMP22, ITPR3
GO:0009891	positive regulation of biosynthetic process	7	0.049	TAF1, DRD1, PDGFB, PRKAA1, SIX4, AHR, PLAGL2
**Cellular component (database: GOTERM_CC_FAT)**				
GO:0005635	nuclear envelope	5	0.008	NUP50, IPO5, TRIM27, AHCTF1, ITPR3
GO:0012505	endomembrane system	8	0.028	AP1S3, DERL2, NUP50, IPO5, TRIM27, AHCTF1, ITPR3, FRS2
GO:0005643	nuclear pore	3	0.037	NUP50, IPO5, AHCTF1
GO:0043233	organelle lumen	13	0.039	TAF1, STOX1, TAF4, PDGFB, TRIM27, AHCTF1, ITPR3, IQGAP1, IPO5, NUP50, TFDP2, SRGN, GTF3C3
GO:0031974	membrane-enclosed lumen	13	0.044	TAF1, STOX1, TAF4, PDGFB, TRIM27, AHCTF1, ITPR3, IQGAP1, IPO5, NUP50, TFDP2, SRGN, GTF3C3
GO:0031981	nuclear lumen	11	0.046	TAF1, TAF4, STOX1, NUP50, IPO5, TFDP2, TRIM27, AHCTF1, ITPR3, IQGAP1, GTF3C3
GO:0005654	nucleoplasm	8	0.050	TAF1, TAF4, NUP50, TFDP2, TRIM27, AHCTF1, ITPR3, GTF3C3
GO:0005667	transcription factor complex	4	0.046	TAF1, TAF4, TFDP2, GTF3C3
**Molecular function (database: GOTERM_MF_FAT)**					
GO:0008095	inositol-1,4,5-trisphosphate receptor activity	2	0.015	CYTH3, ITPR3
GO:0003677	DNA binding	16	0.026	TAF1, ZNF529, STOX1, TAF4, ZBTB34, TRIM27, AHCTF1, SIX4, AHR, HNRNPU, PURA, MYT1L, ZFHX4, TFDP2, GTF3C3, PLAGL2
GO:0005095	GTPase inhibitor activity	2	0.038	IPO5, IQGAP1
GO:0048407	platelet-derived growth factor binding	2	0.042	PDGFB, COL3A1
GO:0008565	protein transporter activity	3	0.044	AP1S3, RAB4A, IPO5

a Gene-enrichment and functional annotation analysis for 79 target genes of miR-4653-3p was performed by using Functional Annotation Tool (DAVID Bioinformatics Resources 6.7, NIAID/NIH, http://david.abcc.ncifcrf.gov/).

b Modified Fisher Exact *P*-Values equal or smaller than 0.05 is considered strongly enriched in the annotation categories than random chance.

### Increased FRS2 expression in recurrent/metastatic lesions versus primary lesions

**Figure 3:**
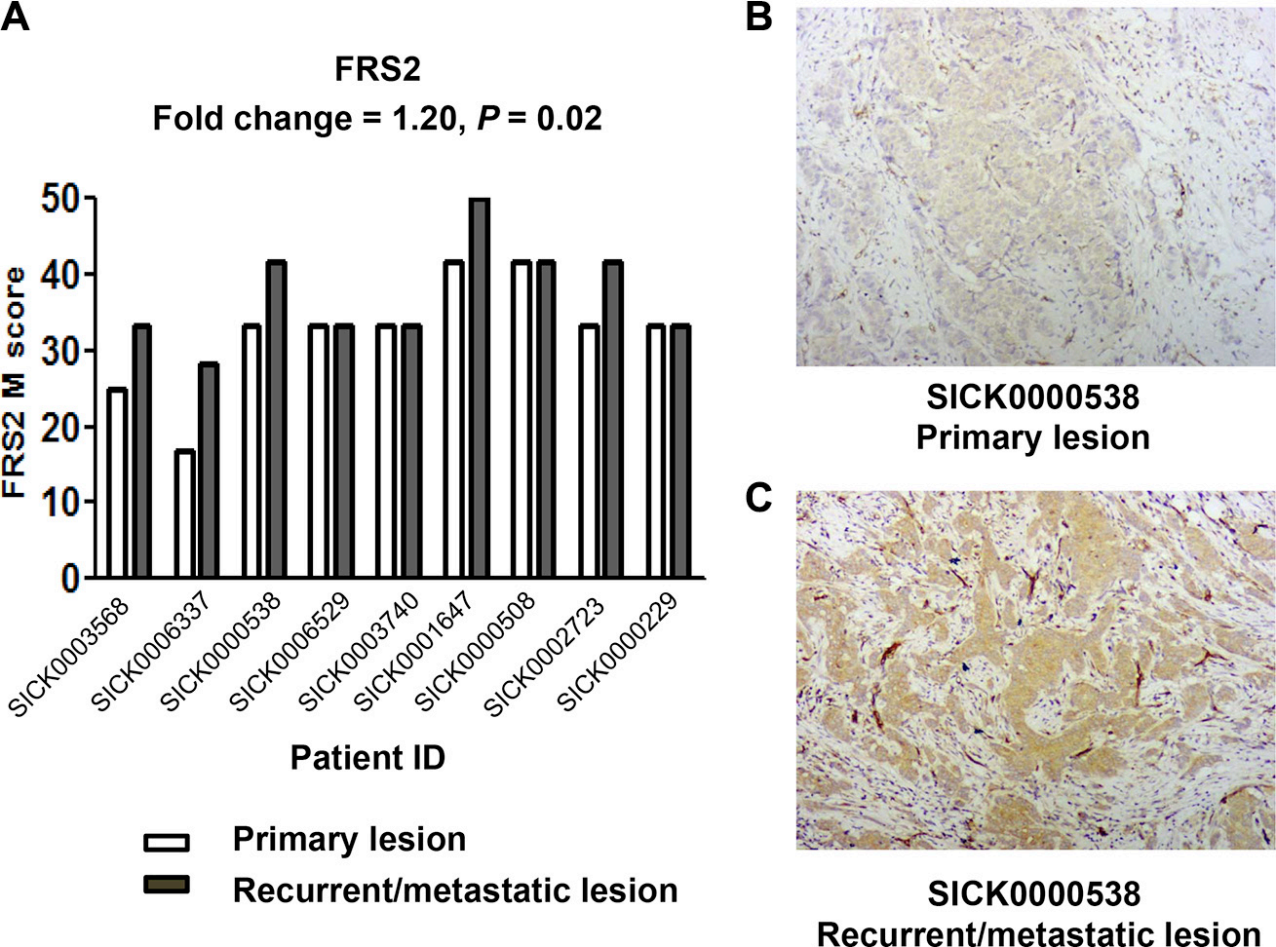
FRS2 was up-regulated in recurrent/metastatic lesions, compared to their matched primary lesions in tamoxifen-resistant patients (**A**) FRS2 expression was compared between paired primary and recurrent/metastatic (R/M) lesions of 9 patients who relapsed after TAM therapy using immunohistochemistry (IHC). M-score was used to estimate FRS2 expression. Fold change was calculated as the mean ratio of M-score in R/M lesions to matched primary lesions. *P* values were calculated using paired two-sided *t*-test. The IHC staining results of (**B**) the primary and (**C**) R/M lesions from Patient SICK0000538 were showed (100X), with an M-score of 33.33 and 41.67, respectively.

FRS2 expression was compared between paired primary and R/M lesions of 9 patients who relapsed after TAM therapy using immunohistochemistry (IHC). There was a slight increase of FRS2 expression in R/M lesions versus primary tumor (FC = 1.20, paired *t*-test *P* = 0.02, Figure 3A). For example, FRS2 M-score for the primary and R/M lesions of Patient SICK0000538 were 33.33 and 41.67, respectively (Figure 3B and 3C).

### FRS2 expression was negatively correlated with miR-4653-3p level and DFS

**Figure 4:**
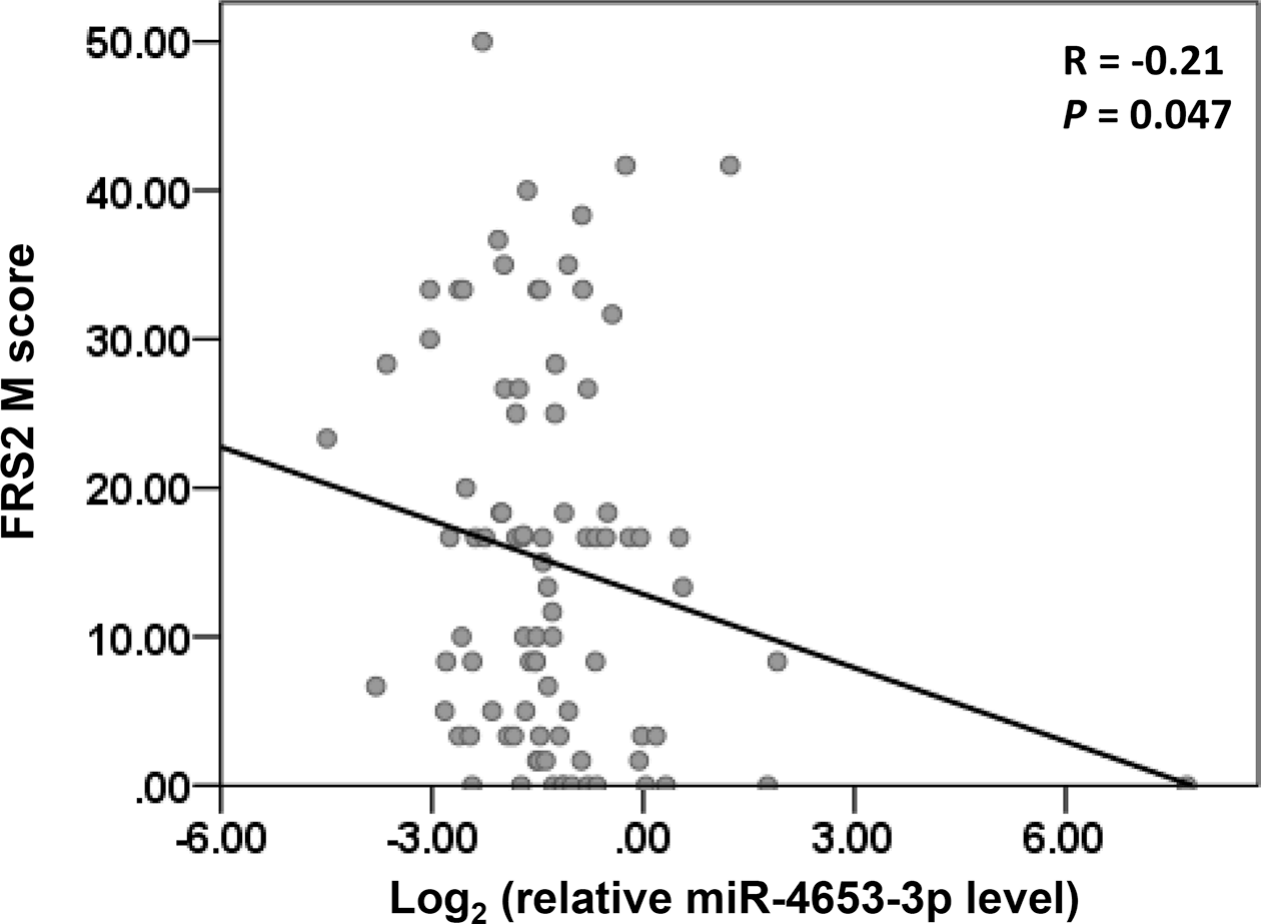
Relative miR-4653-3p level was inversely correlated with FRS2 expression in primary tumor presented as scatter plots *P* value was calculated by Spearman Correlation analysis. Logarithmic-transformed variables of miR-4653-3p level relative to RNU6B were indicated on the X axis.

**Figure 5:**
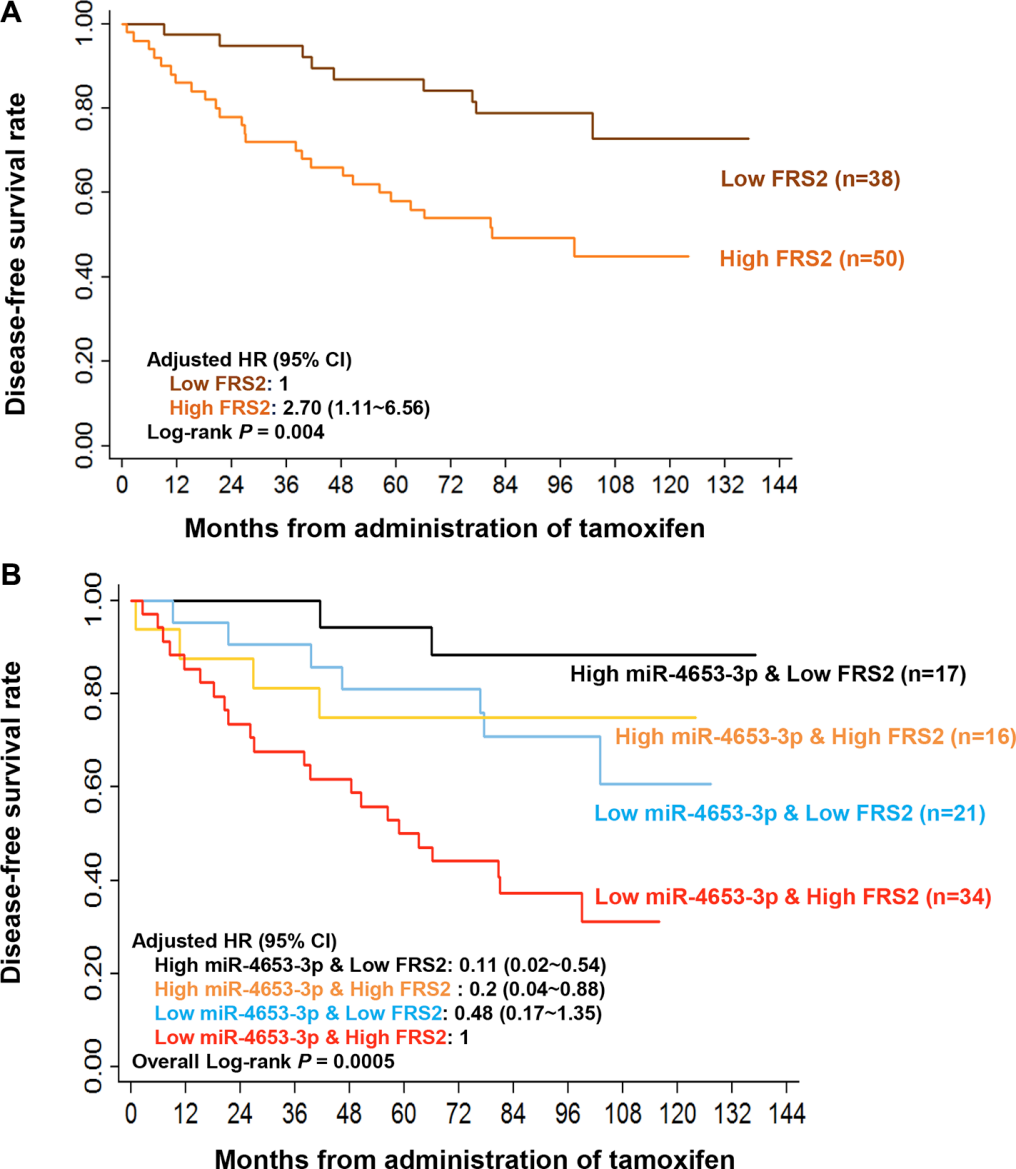
Low miR-4653-3p and high FRS2 expression were associated with poor disease-free survival following tamoxifen treatment (**A**) FRS2 protein expression in primary tumor tissues was detected by IHC. Estimated Kaplan-Meier disease-free survival curve of patients with high (M score ≥ 9.17) and low (M-score < 9.17) were present. The optimal cutoff value for FRS2 expression was determined by ROC curve. (**B**) Estimated Kaplan-Meier disease-free survival curve of breast cancer patients by a combination of miR-4653-3p and FRS2 status were present. Hazard ratio (HR) was adjusted by age at diagnosis, tumor size, lymph node involvement, Ki67 expression, HER2 status, menopause status when receiving tamoxifen and adjuvant chemotherapy. *P* values were calculated using a log-rank analysis.

Next, FRS2 expression was determined in the primary tumors from the validation cohort using IHC. Bivariate correlation analysis showed that FRS2 protein expression was negatively correlated with miR-4653-3p levels to a certain degree (spearman correlation coefficient = −0.21, *P* = 0.047; Figure 4). To define the high and low FRS2 level, we chose a M-score cutoff of 9.17 on the ROC curve for distinguishing patients who were likely to relapse within 5 years. At this cutoff value, the AUC was 0.67 (95% CI = 0.55~0.78, *P* = 0.01) with a sensitivity of 80.8% and specificity of 53.2%. The 5-year DFS rate of high FRS2 expression group (M score ≥ 9.17, *n* = 50) was significantly lower than low expression group (M score < 9.17, *n* = 38) (58.0% ± 7.0% vs. 86.8% ± 5.5%, log-rank *P* = 0.004; Figure 5A). In addition, univariate and multivariate analyses showed that high FRS2 expression was a predictor of poor DFS (unadjusted HR = 2.92; adjusted HR = 2.7, 95% CI = 1.11~6.56, *P* = 0.03; Table 3, Figure 5A).

**Table 3:** Low miR-4653-3p and high FRS2 expression were associated with poor disease-free survival of the validation cohort

Parameter	#Cases (events)	Unadjusted HR (95% CI)^a^	*P*	Adjusted HR (95% CI)^b^	*P*
**FRS2 M score**					
Low (< 9.17)	38 (9)	1		1	
High (≥ 9.17)	50 (26)	2.92 (1.37~6.25)	**0.006**	2.70 (1.11~6.56)	**0.03**
**Combination of miR-4653-3p and FRS2**					
High miR-4653-3p & Low FRS2	17 (2)	0.12 (0.03~0.5)	**0.004**	0.11 (0.02~0.54)	**0.006**
High miR-4653-3p & High FRS2	16 (4)	0.3 (0.1~0.87)	**0.03**	0.2 (0.04~0.88)	**0.03**
Low miR-4653-3p & Low FRS2	21 (7)	0.37 (0.16~0.87)	**0.02**	0.48 (0.17~1.35)	0.16
Low miR-4653-3p & High FRS2	34 (22)	1		1	

a Univariate Cox proportional hazards regression models.

b HR was adjusted by the following confounders: age at diagnosis, tumor size, lymph node involvement, Ki67 expression, HER2 status, menopause status when receiving tamoxifen and adjuvant chemotherapy.

Furthermore, we looked into the predictive potential of a combination of miR-4653-3p and FRS2 status. Low miR-4653-3p & High FRS2 group (50.0% ± 8.6%) had significantly shorter 5-year DFS rate than Low miR- 4653- 3p & Low FRS2 group (81.0% ± 8.6%), High miR-4653-3p & High FRS2 group (75.0% ± 10.8%), and particularly than High miR-4653-3p & Low FRS2 group (94.1% ± 5.7%) (overall log-rank *P* = 0.0005, Figure 5B). High miR- 4653- 3p & low FRS2 group (*n* = 17) had a significantly decreased risk of relapse than Low miR- 4653- 3p & High FRS2 group (*n* = 34) (adjusted HR = 0.11, 95% CI = 0.02~0.54, *P* = 0.006; Table 3, Figure 5B).

### Baseline expression of miR-4653-3p and FRS2 in TAM-resistant cell models

**Figure 6:**
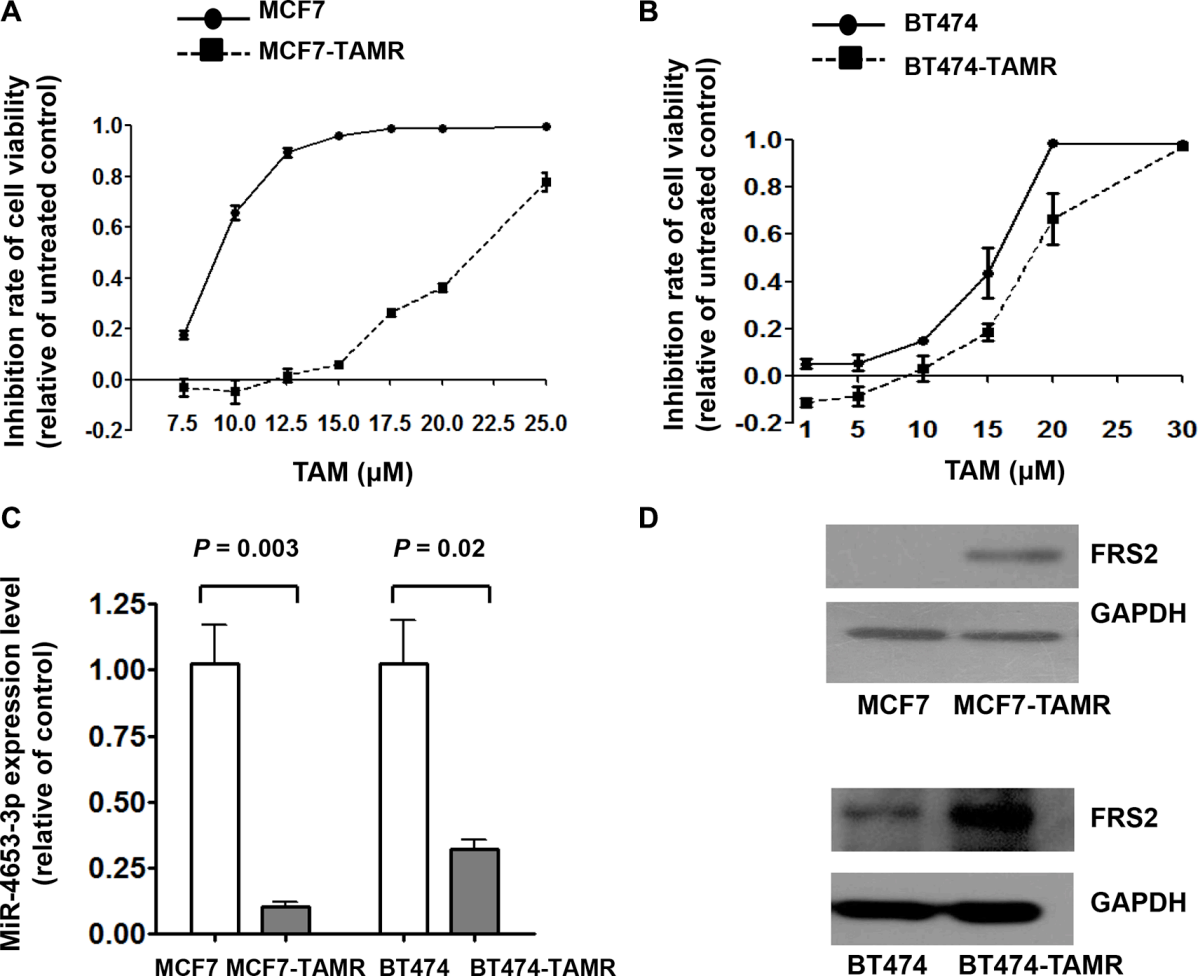
Decrease of miR-4653-3p and increase of FRS2 expression in TAM resistant cell lines compared to parental cells lines (**A**) MCF7-TAMR and MCF7 and (**B**) BT474-TAMR and BT474 cells were grown in complete media in the presence or absence of 4-hydroxy tamoxifen (TAM) of the indicated concentrations for 3 days. Cell viability was detected by MTT assays. Inhibition rate of cell viability relative to untreated controls were represented as mean ± standard deviation. (**C**) Total RNA was isolated from TAM resistant (MCF7-TAMR and BT474-TAMR) and parental cell lines (MCF7 and BT474). Real-time RT-PCR was performed for evaluating miR-4653-3p expression. RNU6B served as an internal control for normalization purpose. (**D**) Cellular protein was isolated from TAM resistant (MCF7-TAMR and BT474-TAMR) and parental cells lines (MCF7 and BT474) followed by Western blot analysis with the antibody against FRS2 protein.

We successfully established two TAM-resistant human breast cancer cell models (MCF7-TAMR and BT474-TAMR). The half maximal inhibitory concentration (IC50) value of TAM in MCF7-TAMR and BT474-TAMR cells were 2.3- and 1.3-fold higher than that of their parental cells, respectively (Figure 6A, 6B). Consistent with the findings in human tumor samples, baseline miR-4653-3p expression levels in MCF7-TAMR and BT474-TAMR cells were 9.8-fold and 3.1-fold lower than the corresponding parental cells, respectively (Figure 6C). In addition, Western blot analysis showed that FRS2 protein expression increased in the two TAM-resistant cell lines (Figure 6D).

### MiR-4653-3p downregulated FRS2 by binding to its 3′ UTR

**Figure 7:**
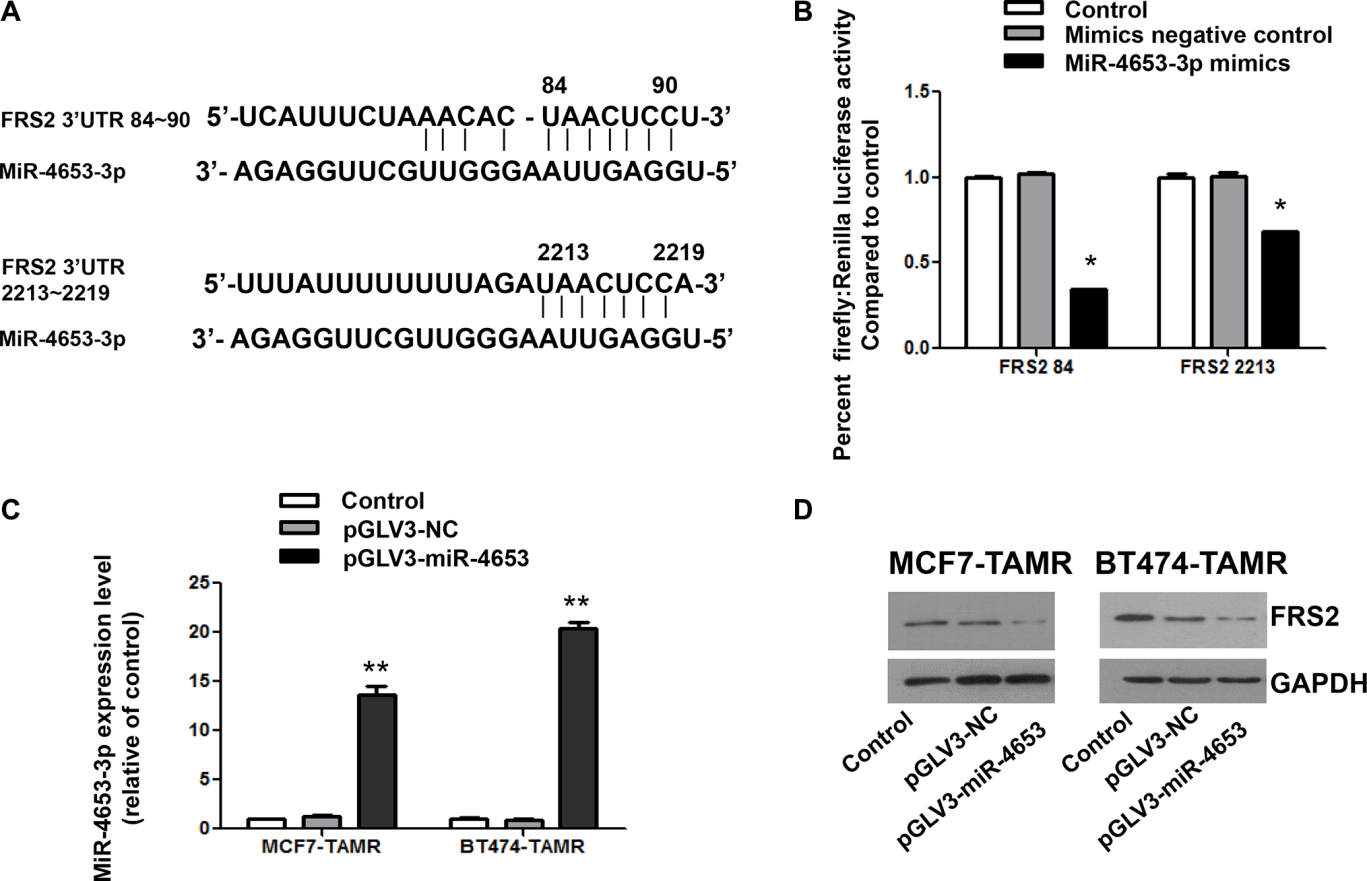
MiR-4653-3p downregulated FRS2 expression by binding to two complimentary sites on 3′UTR of FRS2 mRNA (**A**) Two sites located in 84~90 and 2213~2219 on 3′ UTR of FRS2 mRNA were predicted to be complimentary to miR-4653-3p by multiple databases, miRDB, TargetScan and DIANA. (**B**) 293T cells were transfected with control mimics or miR-4653-3p mimics together with the pmirGLO Vector constructs contained a predicted binding sequence (FRS2 84 or FRS2 2213). Forty-eight hours after transfection, cells were analyzed for luciferase activity using the Dual-Glo^®^ Luciferase Assay System. The bars represent the mean ± standard deviation of at least 3 independent experiments for each condition. * indicates significant decrease of normalized firefly luciferase activity compared to controls and control mimics. *P* < 0.0001 as calculated by One-way ANOVA and LSD test. (**C**) Real-time RT-PCR results for miR-4653-3p level was showed. ** indicates significant overexpression of miR-4653-3p compared to control and pGLV3-NC. *P* < 0.001 as calculated by One-way ANOVA and LSD test. Control, indicates untransfected cells. (**D**) MCF7-TAMR and BT474-TAMR cells were infected with lentivirus particles which mediate miR-4653-3p expression (pGLV3-miR-4653) or the negative control (pGLV3-NC). Western blot results for FRS2 protein were showed.

Human embryonic kidney 293T cells were transfected by pmirGLO Vector constructs containing the predicted binding sites of FRS2 84 (3′ UTR 54~113) or FRS2 2213 (3′ UTR 2184~2243). Dual Luciferase assays showed that overexpression of miR-4653-3p by miRNA mimics downregulated the corresponding luciferase activities by 3.0– and 1.5-fold in transfected 293T cells, as compared to the negative controls (*P* < 0.0001; Figure 7A, 7B). In the TAM-resistant cell lines, lentivirus pGLV3 system was used to overexpress pre-miR-4653. After cleaving and double-strand separation of pre-miRNA, mature miR-4653-3p was generated, as verified by real-time RT-PCR (Figure 7C). Overexpressed miR-4653-3p significantly inhibited FRS2 protein expression in both MCF-TAMR and BT474-TAMR cell lines, as compared to the untransfected control or pGLV3 negative control (Figure 7D). These results suggest that miR-4653- 3p downregulates FRS2 by directly binding to its 3′ UTR.

### MiR-4653-3p increased sensitivity to TAM by downregulating FRS2

**Figure 8:**
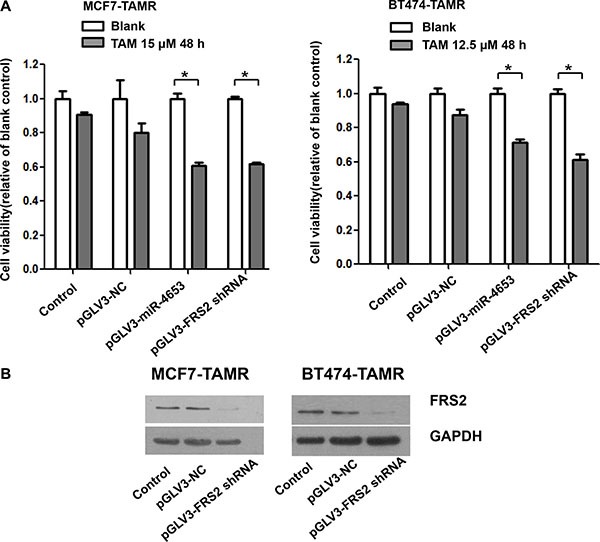
Overexpression of miR-4653-3p and knockdown of FRS2 enhanced the sensitivity to tamoxifen in MCF7-TAMR and BT474-TAMR cells MCF7-TAMR and BT474-TAMR cells were infected with lentivirus particles: pGLV3-miR-4653 which mediates pre-miR-4653 expression, pGLV3-FRS2 shRNA which interferes FRS2 expression, or the negative control (pGLV3-NC). (**A**) Cells were then exposed to TAM (12.5 or 15 μM) for 48 hours. Cell viability was detected by MTT assays. The bars represent the mean ± standard deviation of at least 3 independent experiments for each condition. * indicates significant inhibition of cell viability compared to controls (two-tailed *t*-test *P* < 0.05). (**B**) Cellular protein was isolated from TAM resistant cells followed by Western blot analysis with antibodies against FRS2 protein. GAPDH served as internal control. Control, indicates untransfected cells.

MCF7-TAMR cells infected with pGLV3-NC, pGLV3-miR-4653 or pGLV3-FRS2 shRNA were treated with 15 μM TAM for 48 hours. Relative to the corresponding blank groups, cell viabilities after treatment were 90% and 80% in the uninfected control and pGLV3-NC, respectively. Stable expression of miR-4653-3p by pGLV3-miR-4653 increased the sensitivity of MCF-TAMR to TAM, with a reduced cell viability of 61% after treatment (Figure 8A). Moreover, downregulation of FRS2 expression by pGLV3-FRS2 shRNA (Figure 8B) also reduced the cell viability to 62% after treatment. Similar results were found in the BT474-TAMR cells (Figure 8A, 8B). These data suggested that miR-4653- 3p increased the sensitivity of breast tumor cells to TAM treatment, mostly likely by downregulating FRS2.

## DISCUSSION

To the best of our knowledge, this is the first study to report the prognostic value of miR-4653-3p and its target gene FRS2 in TAM resistance. We identified miR-4653- 3p downregulated in R/M lesions from patients who were refractory to TAM. MiR-4653-3p and FRS2 expression in primary tumor were positively and negative correlated with DFS following TAM adjuvant therapy, respectively. MiR-4653-3p downregulated FRS2 by binding to its 3′ UTR. Either overexpressing miR-4653-3p or attenuating FRS2 expression could restore TAM sensitivity.

Our study adds to the spare literature that utilized human samples in investigating the relationship between miRNAs and TAM resistance [7]. For instance, Hoppe et al. [11] found that increased expression of miR-126 and miR-10a was predictive of prolonged relapse-free time in postmenopausal ER+ BC patients receiving TAM treatment. Jansen et al. [10] found that high miR-26a expression in the primary tumors was associated with longer time to progression in metastatic ER+ BC patients receiving first-line TAM monotherapy. Persson et al. [27] first discovered miR-4653-3p by comparing miRNA expression levels between breast tumor and normal tissues; however, its function remains unknown. In this study, we found that miR-4653-3p decreased by about 2 folds in R/M lesions compared to primary tumors in patients who relapsed following TAM treatment. High miR-4653- 3p level in primary tumor reduced the risk of relapse by 83%. MiR-4653-3p may restore TAM sensitivity by suppressing FRS2 expression in TAM resistant cells. Our findings indicated miR-4653-3p as a promising predictor for favorable outcome of TAM adjuvant therapy.

Although FGFRs are the most studied biomarkers for FGF signaling activation and TAM resistance [17, 28], we pointed out that FRS2, which activating downstream pathways following FGFRs phosphorylation [19], also correlated to TAM resistance. High levels of FGFR3 protein expression [17] as well as FGFR4 mRNA expression [28] were associated with poor prognosis of ER+ breast cancer patients receiving TAM treatment. Exogenous overexpression of PTB domains inhibited FGF-1-induced FRS2 phosphorylation and downstream MAPK and PI3K/AKT/mTOR pathways, leading to decreased colony formation of FGF-1-induced BC cells in an antiestrogen environment [29]. In our study, high FRS2 expression increased the risk of relapse by 2.7 folds in patients receiving TAM adjuvant therapy. High miR-4653- 3p & Low FRS2 group had a decreased risk of relapse than Low miR-4653-3p & High FRS2 group (adjusted HR = 0.11, 95% CI = 0.02~0.54, *P* = 0.006; Table 3, Figure 5B). In comparison, the adjusted HR for high miR-4653-3p level was 0.17 (95% CI = 0.05~0.57, *P* = 0.004; Table 1, Figure 2), and for low FRS2 expression was 0.37 (95% CI = 0.15~0.90, *P* = 0.03; data not showed). It appeared that the correlation between DFS and the combined predictor was similar with that of miR-4653-3p, but stronger than that of FRS2. Our findings suggested that FRS2 overexpression is a promising predictor for poor outcome of TAM adjuvant therapy. A combination predictor of miR-4653-3p and FRS2 may have a stronger predictive power than the separate predictor of FRS2.

Our study had several strengths. The patient selection criteria were close to TAM therapy pattern in the real world. Thereby, patients with HR+ disease regardless of menopause status were the population that could utilize miR-4653-3p and FRS2 as potential predictors. To obtain the reliable biomarkers most relevant to TAM resistance, 5-year duration of treatment was required. The cases receiving AI or other endocrine therapies before the first recurrence were excluded. Findings from functional experiments using TAM-resistant cell models further strengthen the biomarkers' value. Moreover, a relatively long follow-up time allowed us to estimate the prediction power of biomarkers for a long-term survival.

However, the predictive value of miR-4653-3p and FRS2 should be interpretated with caution. We aimed to find prognostic factors especially for TAM treatment, while a majority of patients received both adjuvant chemotherapy and endocrine therapy. This was the case for patients with luminal diseases in the real world study [30]. Moreover, the validation cohort exhibited relatively aggressive clinical characteristics (e.g. 55.7% node positive) with a 5-year relapse rate of 29.5%. In comparison, it was higher than the 5-year recurrence rate (15.4%) of ER+/PR+ patients (45% node positive, 55% chemotherapy) reported previously [3]. Those confounders might increase the possibility to overestimate the predictor's value. To acquire the predictive power of biomarkers independent from other prognostic factors, we adjusted HR by confounders involved in tumor stage, molecular subtype and treatment pattern. Besides, TAM resistance is a complex process involving multiple cell signaling pathways and their cross-talk [31]. To get a comprehensive understanding of TAM resistance, it is worth to explore the roles of other target genes (ex. PDGFB, platelet-derived growth factor beta polypeptide [32]) and other differentially expressed miRNAs related to TAM resistance.

In conclusion, our findings demonstrated that high miR-4653-3p level was the potential predictor for favorable DFS, while FRS2 overexpression was potential high-risk factor for relapse in HR+ BC patients receiving TAM adjuvant therapy. FGFR/FRS2 signaling might be a promising target for reversing TAM resistance.

## MATERIALS AND METHODS

### Study patients and samples

Breast cancer patients were registered in the Breast Cancer Information Management System of West China Hospital, Sichuan University (Sichuan, China) since 1989. Their medical history, pathological diagnosis, and treatment information were prospectively collected by oncologists. Each patient was followed by outpatient visit or telephone at 3 to 4-month intervals within 2 years after diagnosis, 6-month intervals within 3~5 years, and then annually. From March 2001 to September 2013, there were 400 ER+ or PR+ (positive tumor cell ratio > 1%) patients with stage I~III disease, who received TAM as adjuvant therapy continuously for 5 years or until relapse. To avoid the confounding effects, we excluded patients who received AI or other endocrine therapies before the first recurrence. Moreover, FFPE tissue sections were required to contain invasive carcinoma and the tumor cell proportions should be 80% or above. To obtain differentially expressed miRNA profile between paired primary and R/M lesions, we included a discovery set of 6 patients who experienced failure with TAM treatment and with available primary and R/M samples. A total of 100 μm FFPE tissue sections for each sample were processed to miRNA microarray analysis. To investigate the correlation between miR-4653-3p level in primary lesions and DFS following TAM treatment, a validation cohort of 88 patients including 35 cases with relapse was included. A total of 20 μm tissue sections for each sample were processed to real-time RT-PCR for miR-4653-3p detection. Subsequently, FRS2 expression was compared using IHC between paired primary and R/M lesions of 9 patients (5 from the discovery set and 4 from the validation cohort). IHC was also performed in each primary tumor of the validation cohort to determine the association of FRS2 expression and prognosis.

Clinicopathological features of the discovery and validation sets were summarized in Supplementary Table S3 and Table 4, respectively. There were 5 patients present in both sets. Scoring of ER, PR and HER2 were performed according to Guidelines for Testing of ER and PR in Breast Cancer [33] and Guidelines for HER2 Detection in Breast Cancer [34] in China, respectively. Comprehensive therapy was administrated according to National Comprehensive Cancer Network Guidelines (http://www.nccn.org) and St. Gallen International Expert Consensus [35]. Median follow-up time was 149.4 months (range, 78.5~172.2 months) for the discovery set, and 96.4 months (range, 16.2~165.1 months) for the validation cohort. The last follow-up date was Jul 13, 2015. DFS was defined as the interval between beginning of adjuvant TAM therapy and first relapse of cancer, breast cancer-related death, or last follow-up. This study was approved by the Clinical Test and Biomedical Ethics Committee of West China Hospital. Written informed consent was provided by all the patients.

**Table 4:** Clinical and pathological characteristics of the validation cohort of 88 breast cancer patients

Characteristics		Cases	Percentage (%)
Menopause at diagnosis	Premenopause	68	77.3
	Postmenopause	20	22.7
Tumor size	T ≤ 2 cm	21	23.9
	2 cm < T ≤ 5 cm	53	60.2
	T > 5 cm	11	12.5
	Unknown	3	3.4
Lymph node involvement	Negative	38	43.2
	Positive	49	55.7
	Unknown	1	1.1
Clinical stage	I	17	19.3
	II	41	46.6
	III	27	30.7
	Unknown	3	3.4
Tumor histology	Invasive ductal carcinoma	80	90.9
	Other invasive carcinoma ^a^	8	9.1
Tumor grade	I/II	35	39.8
	III	48	54.5
	Unknown	5	5.7
Molecular subtype	Luminal A	24	27.3
	Luminal B	49	55.7
	Unknown	15	17.0
ER	Negative	8	9.1
	Positive	80	90.9
PR	Negative	8	9.1
	Positive	80	90.9
Ki67	< 14%	33	37.5
	≥ 14%	49	55.7
	Unknown	6	6.8
HER2	Negative	76	86.4
	Positive	5	5.7
	Uncertain	7	8.0
Menopause when receiving tamoxifen	Premenopause	63	71.6
	Postmenopause	23	26.1
	Unknown	2	2.3
Adjuvant chemotherapy	No	4	4.5
	Yes	84	95.5
Adjuvant radiotherapy	No	43	48.9
	Yes	45	51.1
**Characteristics**		**Median (range)**	
Age at diagnosis (year)		45 (25~71)					
Months of tamoxifen adminstration by relapse		60.9 (1.0~137.1)					
Follow-up months after tamoxifen administration		96.4 (16.2~165.1)					

a Mucinous adenocarcinoma, invasive lobular carcinoma, invasive micropapillary carcinoma, and mixed type carcinoma.

### MicroRNA microarray

Total RNA was extracted from deparaffinized FFPE tissue sections using TRIzol (Invitrogen, Carlsbad, CA, USA) and miRNeasy mini kit (QIAGEN, Hilden, Germany) according to manufacturer's instructions. RNA quantity was measured using NanoDrop 1000 (Nanodrop Technologies). The samples were 3′-end-labeled with Hy3^TM^ using miRCURY™ Power labeling kit (Exiqon, Vedbaek, Denmark) and hybridized on the miRCURY™ LNA Array (v.18.0) (Exiqon, Vedbaek, Denmark). The slides were scanned using the Axon GenePix 4000B microarray scanner (Axon Instruments, Foster City, CA) and then analyzed by Axon GenePix Pro 6.0 software (Axon Instruments). Expressed data were normalized using the Median normalization. Shapiro-Wilk test using open source software R, paired *t*-test and Mann–Whitney *U*-test in Matlab computation environment were performed to determine statistical significance of differentially expressed miRNAs.

### Real-time reverse transcription polymerase chain reaction (RT-PCR)

Total RNA was isolated from deparaffinized FFPE tissue sections using miRNeasy FFPE Kit (Qiagen), or from cells using TRIzol (Invitrogen) according to the manufacturer's instructions. Real-time RT-PCR reaction was performed using All-in-One^TM^ miRNA qRT-PCR Detection Kit (GeneCopoeia, USA) on a DNA Engine Peltier Thermal Cycler Chromo4 (Bio-Rad). Primers for miR-3687 (HmiRQP1972), miR-4653-3p (HmiRQP2251), miR-144-3p (HmiRQP0190), miR- 660- 5p (HmiRQP0771), and RNU6B (HmiRQP9001) were obtained from GeneCopoeia. RNU6B, which was reported to be unaffected by hormone treatment, served as the normalization control [36]. Relative quantification was performed using the 2^−ΔΔ^CT method.

### Immunohistochemistry of FRS2 expression

IHC was performed using FFPE tissue slides and Elivision^TM^ super HRP (Mouse/Rabbit) IHC Kit (Fuzhou Maixin Biotech, China) according to the manufacturer's instructions. After deparaffinization and rehydration, the slides were incubated at 97°C with Target Retrieval Solution pH 9 (EDTA, pH 9, Gene Tech, Shanghai, China) for 40 min. An overnight incubation with rabbit polyclonal antibody against FRS2 (dilution 1:100, Santa Cruz biotechnology, USA) at 4°C was performed. Unspecific peroxidase activity was blocked by 3% H_2_O_2_. After wash, the slides were incubated with Amplifier for 15 min and subsequently with Peroxidase-labeled polymer conjugated to goat anti-mouse/rabbit immunoglobulin for another 15 min at room temperature. Following this, the slides were developed with a 3,3′-diaminobenzidine tetrahydrochloride (DAB) solution and counter-stained with hematoxylin. Vascular endothelial cells served as internal positive controls and phosphate buffer saline (PBS) as the blank control. Scoring for staining was performed by a senior pathologist. M-score system was used to weigh both positive cell proportion and staining intensity, as described previously [37]. IHC membrane and cytoplasmic staining intensities of FRS2 were scored as: -, no staining; 1+, weak; 2+, moderate, and 3+, strong (Supplementary Figure S1).

### Cell culture and chemicals

HR+ human BC cell lines (MCF7 and BT474) were obtained from the American Type Culture Collection (Manassas, VA, USA). MCF7 and BT474 cells were routinely grown in RPMI-1640 and DMEM medium, respectively, supplemented with 10% fetal bovine serum, 2 mM glutamine, 4-(2-hydroxyethyl)-1-piperazineethanesulfonic acid (HEPES) buffer, and 100 unit/ml penicillin/streptomycin. Additionally, 0.1 unit/ml insulin was added to the culture medium for BT474. 4-hydroxy TAM was purchased from Sigma-Aldrich, USA. The TAM-resistant cell lines, MCF7-TAMR and BT474-TAMR, were generated by continuous exposure of MCF7 and BT474 cells to increasing doses of TAM up to 13 μM in 6 months and thereafter maintaining them in presence of 13 μM TAM. TAM resistant cells were cultured in the absence of TAM for 3 days to avoid the influence of TAM in subsequent experiments. Rabbit polyclonal antibody against FRS2 and mouse monoclonal antibody against GAPDH were obtained from Santa Cruz biotechnology, USA.

### Western blot analysis

The procedure was performed as previously described [38] Briefly, equal amounts of protein from whole cell lysates were separated on 10% SDS polyacrylamide gels and blotted onto PVDF membrane. Blots were incubated with antibodies against FRS2 and GAPDH over night, followed by incubation with secondary goat antibody raised against rabbit or mouse immunoglobin conjugated to horseradish peroxidase (Zen Bioscience, Chengdu, China). The bands were visualized using Immobilon Western Chemiluminescence HPR Substrate (Millpore corp., Billerica, MA, USA).

### FRS2 3′UTR construct and dual luciferase assay

Two fragments containing predicted binding sites, FRS2 84 (NM_001042555.2 3′ UTR 54~113) and FRS2 2213 (NM_001042555.2 3′ UTR 2184~2243), were cloned into pmirGLO Vectors (Promega, Madison, WI) by GenePhama (Shanghai, China). The primers used were listed in Supplementary Table S4. The sequences of the resulting vectors were verified by sequencing. Human embryonic kidney 293T cells (Cell Bank of Chinese Academy of Science, Beijing, China) were pre-seeded in a 24-well plate at the density of 1 × 10^5^ cells/well and cultured for 24 hours. Next, 293T cells were co-transfected with miR-4653-3p mimics or control mimics (final concentration 100 nM, GenePharma, Shanghai, China) together with pmirGLO Vector constructs (0.8 μg/well) using the Lipofectamine 2000 reagent (Invitrogen, Carlsbad, CA, USA) according to the manufacturer's instructions. Forty-eight hours after transfection, Firefly luciferase and Renilla activity was assayed using the Dual-Glo^®^ Luciferase Assay System (Promega).

### Plasmid construction, lentivirus packaging and cell infection

The shRNA templates which express pre-miR-4653 or interfer FRS2 expression were constructed into a lentiviral vector, pGLV3/H1/green fluorescent protein+Puro (pGLV3; GenePhama). The primers used for generating templates were listed in Supplementary Table S4. Positive clones were picked and verified by DNA sequencing. The pGLV3 vectors and packing plasmids (GenePhama) were co-transfected using RNAi-mate (GenePhama) into 293T cells, according to the manufacturer's instruction. After 72 hours, the supernatant was harvested. The packaged lentiviruses were termed pGLV3-miR-4653, pGLV3-FRS2 shRNA. PGLV3-NC was used as a negative control. MCF7-TAMR and BT474-TAMR cells were infected with the above lentivirus particles (multiplicity of infection = 10) in the presence of 5 μg/ml polybrene (GenePhama). After reaching confluence, the infected cells were selected with fresh medium containing 5 μg/ml puromycin for 4~5 passages. TAM resistant cells stably expressing miR-4653- 3p or with a lower expression of FRS2 were generated and eventually cryopreserved for later use.

### Cell viability assay

TAM resistant and the parental cell lines were plated at the indicated densities (3000 cells/well) in 96-well culture plates (Costar, Cambridge, MA, USA) and exposed to TAM at different concentrations for 2~3 days. Cell viability was evaluated by MTT assays (Sigma-Aldrich, USA) according to the manufacturer's instructions.

### Statistical analysis

Paired *t*-test and Wilcoxon signed ranks test (related samples) were performed to compare relative miRNA level and FRS2 expression between primary and R/M lesions as appropriate. ROC curve was used to determine the optimal cutoff point for the expression levels of miR-4653-3p and FRS2 to predict a poor 5-year DFS with maximum sensitivity and specificity. The log-rank test and Kaplan-Meier plots were used to visualize survival characteristics (STATA version 12; StataCorp, College Station, TX). Survival analyses were performed using univariate and multivariate Cox proportional hazards regression models (SPSS version 20, IBM corp., USA). Spearman bivariate correlation analysis was used to calculate the correlation between miR-4653-3p and FRS2 expression. Probit analysis was used to calculate IC50 of TAM in breast cancer cells (SPSS version 20). All experiments were repeated a minimum of 3 times. All data were expressed as means ± standard deviation. Statistical significance was determined with Student's *t* test (two-tailed) comparison between 2 groups of data sets. One-way analysis of variance (ANOVA) was used for comparison among 3 or more groups. A two-sided test *P* value < 0.05 was judged as statistically significant.

## SUPPLEMENTARY MATERIALS FIGURES AND TABLES






