# Supplementary material for: MiR-4653-3p and its target gene FRS2 are prognostic biomarkers for hormone receptor positive breast cancer patients receiving tamoxifen as adjuvant endocrine therapy
Source: Oncotarget. 2016 Aug 13;7(38):61166–82. doi: 10.18632/oncotarget.11278 (PMC5308643; doi:10.18632/oncotarget.11278)
Supplement: Supplementary file 1 [file oncotarget-07-61166-s001.docx]

**Table S1. Comparison of miRNA expression profiles of paired primary and recurrent/metastatic lesions from the discovery set.**

| **MiRNA name** | **Fold change ^a^** | ***P* ^b^** |
| --- | --- | --- |
| **28 down-regulated miRNAs compared to primary tumor** | | |
| hsa-miR-3687 | 0.27 | 0.006 |
| hsa-miR-4653-3p | 0.28 | 0.03 |
| hsa-miR-4694-3p | 0.31 | 0.02 |
| hsa-miR-4501 | 0.34 | 0.004 |
| hsa-miR-4535 | 0.35 | 0.03 |
| hsa-miR-3648 | 0.36 | 0.003 |
| hsa-miR-3178 | 0.37 | 0.02 |
| hsa-miR-125b-1-3p | 0.38 | 0.008 |
| hsa-miR-3177-5p | 0.39 | 0.04 |
| hsa-miR-4433-3p | 0.39 | 0.04 |
| hsa-miR-4429 | 0.41 | 0.004 |
| hsa-miR-4783-3p | 0.41 | 0.008 |
| hsa-miR-378h | 0.43 | 0.01 |
| hsa-miR-4507 | 0.43 | 0.02 |
| hsa-miR-4530 | 0.44 | 0.02 |
| hsa-miR-650 | 0.45 | 0.02 |
| hsa-miR-4497 | 0.45 | 0.03 |
| hsa-miR-4690-5p | 0.45 | 0.005 |
| hsa-miR-4706 | 0.46 | 0.05 |
| hsa-miR-711 | 0.46 | 0.02 |
| hsa-miR-4314 | 0.46 | 0.002 |
| hsa-miR-516b-5p | 0.47 | 0.03 |
| hsa-miR-1285-3p | 0.47 | 0.01 |
| hsa-miR-1184 | 0.47 | 0.02 |
| hsa-miR-3606-5p | 0.48 | 0.01 |
| hsa-miR-4426 | 0.49 | 0.02 |
| hsa-miR-665 | 0.49 | 0.04 |
| hsa-miR-5698 | 0.50 | 0.02 |
| **54 up-regulated miRNAs compared to primary tumor** | | |
| hsa-miR-191-5p | 13.63 | 0.05 |
| hsa-miR-451a | 10.69 | 0.03 |
| hsa-miR-144-3p | 10.27 | 0.04 |
| hsa-miR-519a-3p | 5.63 | 0.03 |
| hsa-miR-571 | 5.05 | 0.04 |
| hsa-miR-3607-3p | 4.91 | 0.04 |
| hsa-miR-609 | 4.67 | 0.04 |
| hsa-miR-532-5p | 4.49 | 0.04 |
| hsa-miR-539-5p | 4.45 | 0.04 |
| hsa-miR-3913-5p | 4.36 | 0.02 |
| hsa-miR-676-3p | 4.33 | 0.04 |
| hsa-miR-365a-3p/hsa-miR-365b-3p | 4.27 | 0.03 |
| hsa-miR-155-5p | 4.24 | 0.05 |
| hsa-miR-204-5p | 4.06 | 0.04 |
| hsa-miR-432-5p | 4.06 | 0.04 |
| hsa-miR-3192 | 4.03 | 0.04 |
| hsa-miR-1262 | 4.01 | 0.006 |
| hsa-miR-4649-3p | 3.92 | 0.02 |
| hsa-miR-147a | 3.91 | 0.04 |
| hsa-miR-1207-5p | 3.74 | 0.04 |
| hsa-miR-660-5p | 3.70 | 0.004 |
| hsa-miR-4281 | 3.64 | 0.04 |
| hsa-miR-502-3p | 3.64 | 0.04 |
| hsa-miR-193b-3p | 3.53 | 0.03 |
| hsa-miR-499a-5p | 3.49 | 0.02 |
| hsa-miR-1228-5p | 3.40 | 0.04 |
| hsa-miR-211-5p | 3.39 | 0.04 |
| hsa-miR-627 | 3.30 | 0.001 |
| hsa-miR-507 | 3.22 | 0.04 |
| hsa-miR-24-1-5p | 3.17 | 0.04 |
| hsa-miR-1237-3p | 3.12 | 0.03 |
| hsa-miR-139-5p | 3.10 | 0.04 |
| hsa-miR-338-5p | 3.03 | 0.01 |
| hsa-miR-1225-3p | 3.02 | 0.03 |
| hsa-miR-323a-5p | 2.81 | 0.04 |
| hsa-miR-4652-3p | 2.81 | 0.04 |
| hsa-miR-522-3p | 2.80 | 0.04 |
| hsa-miR-202-5p | 2.80 | 0.02 |
| hsa-miR-642a-5p | 2.62 | 0.04 |
| hsa-miR-331-3p | 2.60 | 0.05 |
| hsa-miR-3170 | 2.54 | 0.01 |
| hsa-miR-92a-1-5p | 2.49 | 0.04 |
| hsa-miR-432-3p | 2.48 | 0.04 |
| hsa-miR-376a-5p | 2.42 | 0.002 |
| hsa-miR-324-5p | 2.34 | 0.01 |
| hsa-miR-718 | 2.29 | 0.05 |
| hsa-miR-5681b | 2.24 | 0.03 |
| hsa-miR-3679-3p | 2.21 | 0.05 |
| hsa-miR-431-5p | 2.16 | 0.03 |
| hsa-miR-22-5p | 2.14 | 0.03 |
| hsa-miR-612 | 2.14 | 0.006 |
| hsa-miR-371b-3p | 2.07 | 0.01 |
| hsa-let-7b-3p | 2.04 | 0.05 |
| hsa-miR-1244 | 2.00 | 0.04 |

^a^ Fold change was calculated as the mean ratio of normalized miRNA levels of recurrent/metastatic lesions to matched primary lesions.

^b^ *P*-value calculated by paired two-tailed t-tests, or Mann–Whitney U-test for 2-related-samples as appropriate.
