# Supplementary material for: MiR-4653-3p and its target gene FRS2 are prognostic biomarkers for hormone receptor positive breast cancer patients receiving tamoxifen as adjuvant endocrine therapy
Source: Oncotarget. 2016 Aug 13;7(38):61166–82. doi: 10.18632/oncotarget.11278 (PMC5308643; doi:10.18632/oncotarget.11278)
Supplement: Supplementary file 2 [file oncotarget-07-61166-s002.docx]

**Table S2. Overlap of predicted target genes of miR-4653-3p by 3 databases (miRDB, TargetScan and DIANA) ^a^.**

| **Gene symbol** | **Gene name** |
| --- | --- |
| PPP2CA | protein phosphatase 2, catalytic subunit, alpha isozyme |
| SRSF4 | serine/arginine-rich splicing factor 4 |
| TBC1D12 | TBC1 domain family, member 12 |
| IGF2BP2 | insulin-like growth factor 2 mRNA binding protein 2 |
| TM9SF4 | transmembrane 9 superfamily protein member 4 |
| SEPT7 | septin 7 |
| IPO5 | importin 5 |
| CADM2 | cell adhesion molecule 2 |
| WDR11 | WD repeat domain 11 |
| AHCTF1 | AT hook containing transcription factor 1 |
| MYT1L | myelin transcription factor 1-like |
| TTC37 | tetratricopeptide repeat domain 37 |
| SAMD9 | sterile alpha motif domain containing 9 |
| NIPBL | Nipped-B homolog (Drosophila) |
| ACTR1A | ARP1 actin-related protein 1 homolog A, centractin alpha (yeast) |
| SEMA6D | sema domain, transmembrane domain (TM), and cytoplasmic domain, (semaphorin) 6D |
| FRS2 | fibroblast growth factor receptor substrate 2 |
| CYTH3 | cytohesin 3 |
| FAM171A1 | family with sequence similarity 171, member A1 |
| MYRF | myelin regulatory factor |
| PTPRB | protein tyrosine phosphatase, receptor type, B |
| ZNF529 | zinc finger protein 529 |
| DMD | dystrophin |
| KIAA0355 | KIAA0355 |
| SMEK2 | SMEK homolog 2, suppressor of mek1 (Dictyostelium) |
| TAF1 | TAF1 RNA polymerase II, TATA box binding protein (TBP)-associated factor, 250kDa |
| NDUFB5 | NADH dehydrogenase (ubiquinone) 1 beta subcomplex, 5, 16kDa |
| ZFHX4 | zinc finger homeobox 4 |
| CCBE1 | collagen and calcium binding EGF domains 1 |
| HNRNPU | heterogeneous nuclear ribonucleoprotein U (scaffold attachment factor A) |
| TSLP | thymic stromal lymphopoietin |
| GDA | guanine deaminase |
| NFASC | neurofascin |
| PLAGL2 | pleiomorphic adenoma gene-like 2 |
| COL3A1 | collagen, type III, alpha 1 |
| UBE2W | ubiquitin-conjugating enzyme E2W (putative) |
| ITPR3 | inositol 1,4,5-trisphosphate receptor, type 3 |
| KRT40 | keratin 40 |
| MAGEB6 | melanoma antigen family B, 6 |
| GOLT1B | golgi transport 1B |
| DERL2 | derlin 2 |
| AP1S3 | adaptor-related protein complex 1, sigma 3 subunit |
| TAF4 | TAF4 RNA polymerase II, TATA box binding protein (TBP)-associated factor, 135kDa |
| TMEM200A | transmembrane protein 200A |
| PURA | purine-rich element binding protein A |
| RPL37 | ribosomal protein L37 |
| SYT15 | synaptotagmin XV |
| TAGAP | T-cell activation RhoGTPase activating protein |
| SRBD1 | S1 RNA binding domain 1 |
| AHR | aryl hydrocarbon receptor |
| TXNDC16 | thioredoxin domain containing 16 |
| TRIM27 | tripartite motif containing 27 |
| NUP50 | nucleoporin 50kDa |
| IQGAP1 | IQ motif containing GTPase activating protein 1 |
| STIM2 | stromal interaction molecule 2 |
| TFDP2 | transcription factor Dp-2 (E2F dimerization partner 2) |
| RAB4A | RAB4A, member RAS oncogene family |
| ZBTB34 | zinc finger and BTB domain containing 34 |
| KMT2A | lysine (K)-specific methyltransferase 2A |
| ABHD4 | abhydrolase domain containing 4 |
| BZW1 | basic leucine zipper and W2 domains 1 |
| GTF3C3 | general transcription factor IIIC, polypeptide 3, 102kDa |
| PHYHIPL | phytanoyl-CoA 2-hydroxylase interacting protein-like |
| DRD1 | dopamine receptor D1 |
| MLLT11 | myeloid/lymphoid or mixed-lineage leukemia (trithorax homolog, Drosophila); translocated to, 11 |
| STOX1 | storkhead box 1 |
| PDGFB | platelet-derived growth factor beta polypeptide |
| TSTD2 | thiosulfate sulfurtransferase (rhodanese)-like domain containing 2 |
| RBM43 | RNA binding motif protein 43 |
| AHCYL2 | adenosylhomocysteinase-like 2 |
| SRGN | serglycin |
| RNF44 | ring finger protein 44 |
| PRKAA1 | protein kinase, AMP-activated, alpha 1 catalytic subunit |
| SIX4 | SIX homeobox 4 |
| FZD4 | frizzled family receptor 4 |
| ZYG11B | zyg-11 family member B, cell cycle regulator |
| PCDHB3 | protocadherin beta 3 |
| S100A3 | S100 calcium binding protein A3 |
| PMP22 | peripheral myelin protein 22 |

^a^ MiR-4653-3p-targeted genes were predicted in 3 databases: 144 in miRDB (MirTarget2, http://mirdb.org/miRDB/), 3221 in TargetScan (TargetScan7.1, http://www.targetscan.org/) and 530 in DIANA (MICROT MicroT-CDS, http://diana.imis.athena-innovation.gr/DianaTools/index.php). The 79 common predicted genes were listed here.
