# Supplementary material for: MiR-4653-3p and its target gene FRS2 are prognostic biomarkers for hormone receptor positive breast cancer patients receiving tamoxifen as adjuvant endocrine therapy
Source: Oncotarget. 2016 Aug 13;7(38):61166–82. doi: 10.18632/oncotarget.11278 (PMC5308643; doi:10.18632/oncotarget.11278)
Supplement: Supplementary file 3 [file oncotarget-07-61166-s003.pdf]

MiR-4653-3p and its target gene FRS2 are prognostic biomarkers for hormone receptor positive breast cancer patients receiving tamoxifen as adjuvant endocrine therapy

Supplementary Materials

Supplementary Table S1: Comparison of miRNA expression profiles of paired primary and recurrent/metastatic lesions from the discovery set. [a]Fold change was calculated as the mean ratio of normalized miRNA levels of recurrent/metastatic lesions to matched primary lesions. [b]*P*-value calculated by paired two-tailed *t*-tests, or Mann–Whitney *U*-test for 2-related-samples as appropriate. See Supplementary_Table_S1

Supplementary Table S2: Overlap of predicted target genes of miR-4653-3p by 3 databases (miRDB, TargetScan and DIANA)[a]. [a]MiR-4653-3p-targeted genes were predicted in 3 databases: 144 in miRDB (MirTarget2, http://mirdb.org/miRDB/), 3221 in TargetScan (TargetScan7.1, http://www.targetscan.org/) and 530 in DIANA (MICROT MicroT-CDS, http://diana.imis.athena-innovation.gr/DianaTools/index.php). The 79 common predicted genes were listed here. See Supplementary_Table_S2

Supplementary Table S3: Clinical characteristics of the discovery set of 6 breast cancer patients

Characteristics		Patient No.	
Menopause at diagnosis	Premenopause	4	
	Postmenopause	2	
Tumor size	T ≤ 2 cm	1	
	2 cm < T ≤ 5 cm	3	
	T > 5 cm	1	
	Unkown	1	
Lymph node involvement	Negative	0	
	Positive	6	
Clincal stage	II	1	
	III	4	
	Unkown	1	
Tumor histology	Invasive ductal carcinoma	6	
Tumor grade	I/II	4	
	III	2	
		Primary lesion	Recurrent/metastatic lesion
ER	Negative	1	0
	Positive	5	6
PR	Negative	0	1
	Positive	6	5
Ki67	< 14%	3	3
	≥ 14%	3	3
HER2	Negative	4	4
	Positive	1	0
	Uncertain	1	2
Menopause when receiving tamoxifen	Premenopause	4	
	Postmenopause	2	
Adjuvant chemotherapy	No	0	
	Yes	6	
Adjuvant radiotherapy	No	2	
	Yes	4	

Characteristics	Median (range)
Age at diagnosis (year)	40.5 (34~56)
Months of tamoxifen adminstration by relapse	49.6 (26.2~60.9)
Follow-up months after tamoxifen administration	149.4 (78.5~172.2)

Supplementary Table S4: Primer sequences used in constructions of pmirGLO Vectors and lentiviral vectors

Primer sequences (5′-3′)	
**FRS2 84 (NM_001042555.2 3’UTR 54-113) constructed into pmirGLO Vectors**	
**Sense**	CTGCAAGTGCTTCATTTTCATTTCTAAACACTAACTCCTTTTATAGACTGATAAAATTTTTC
**Antisense**	TCGAGAAAAATTTTATCAGTCTATAAAAGGAGTTAGTGTTTAGAAATGAAAATGAAGCAC TTGCAGAGCT
**FRS2 2213 (NM_001042555.2 3’UTR 2184-2243) constructed into pmirGLO Vectors**	
**Sense**	CGATTCTAATTAGTTTTTATTTTTTTTAGATAACTCCAATATAATCATTACAGTTTATGCTC
**Antisense**	TCGAGAGCATAAACTGTAATGATTATATTGGAGTTATCTAAAAAAAATAAAAACTAATTAGA ATCGAGCT
**FRS2 shRNA constructed into pGLVH1/GFP+Puro**	
**Sense**	GATCCGCTGCTCAGAACTTACCTAATTTCAAGAGAATTAGGTAAGTTCTGAGCAGCTTTTTTG
**Antisense**	AATTCAAAAAAGCTGCTCAGAACTTACCTAATTCTCTTGAAATTAGGTAAGTTCTGAGCAGCG
**Hsa-miR-4653-pre constructed into pGLVH1/GFP+Puro**	
**Sense**	GATCCTTGTCCAATTCTCTGAGCAAGGCTTAACACCAAAGGGTTAAGGGTTTGCTCTGGAGTT AAGGGTTGCTTGGAGAATTGGAGAAG
**Antisense**	AATTCTTCTCCAATTCTCCAAGCAACCCTTAACTCCAGAGCAAACCCTTAACCCTTTGGTGTTAA GCCTTGCTCAGAGAATTGGACAAG

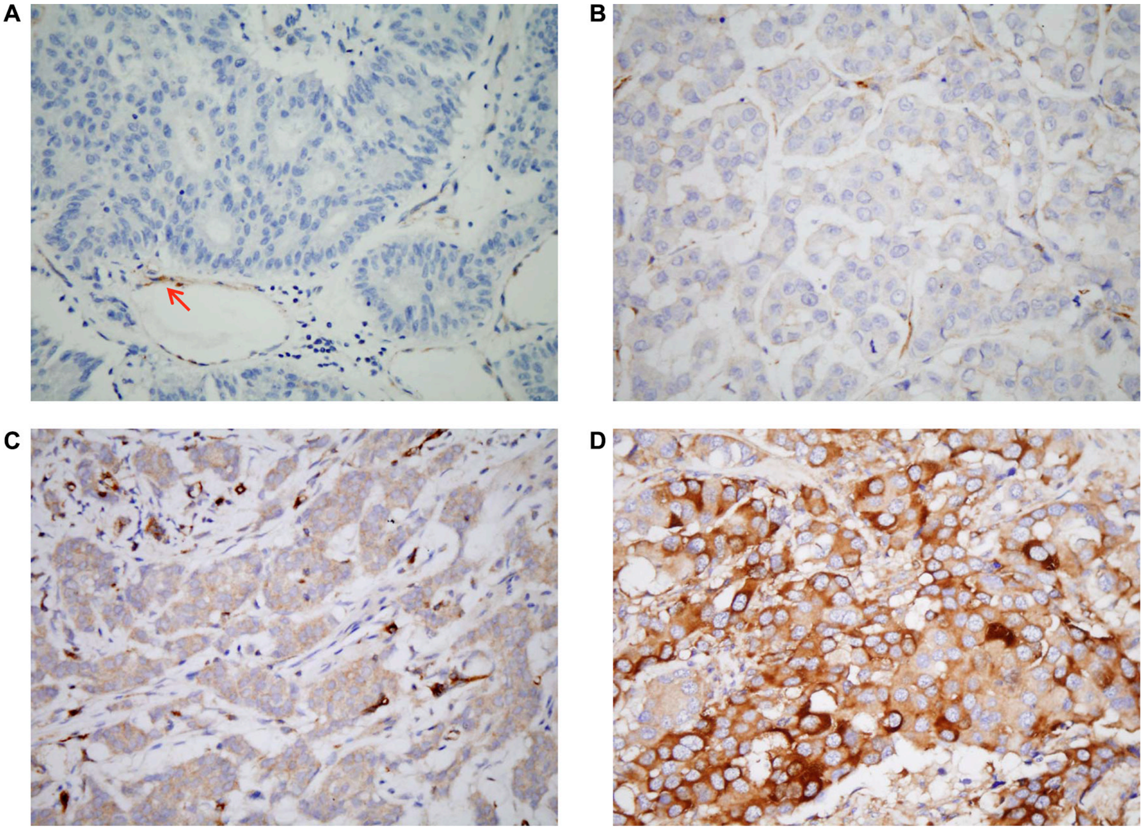

Supplementary Figure S1: IHC scoring of FRS2 expression in invasive breast carcinoma samples (400X). IHC membrane and cytoplasmic staining intensities of FRS2 were scored as: (**A**), −, no staining; (**B**), 1+, weak; (**C**), 2+, moderate, and (**D**), 3+, strong. The M-score was used to analyze the expression level of FRS2 protein, which weighs both positive cell proportion and staining intensity. For example, if 40% of tumor cells stained with intensity 3+, 60% with intensity 2+, and none with intensity +, $M = (40 \times 3 + 60 \times 2 + 0 \times 1)/6 = 40.00$; If 50% of tumor stained with intensity +, and 50% with no staining, $M = (0 \times 3 + 0 \times 2 + 50 \times 1)/6 = 8.33$. Vascular endothelial cells served as internal positive controls (red arrow).
